# Natural Active Ingredients for Diabetes and Metabolism Disorders Treatment

**DOI:** 10.1155/2016/2965214

**Published:** 2016-11-06

**Authors:** Hilal Zaid, Abbas A. Mahdi, Akhilesh K. Tamrakar, Bashar Saad, Mohammed S. Razzaque, Amitava Dasgupta

**Affiliations:** ^1^Qasemi Research Center, Al-Qasemi Academic College, P.O. Box 124, 30100 Baqa Al-Gharbiyye, Israel; ^2^Faculty of Arts and Sciences, Arab American University Jenin, P.O. Box 240, Jenin, State of Palestine; ^3^Department of Biochemistry, King George's Medical University, Lucknow, India; ^4^Division of Biochemistry, CSIR-Central Drug Research Institute, Lucknow, India; ^5^Department of Applied Oral Sciences, The Forsyth Institute, Harvard School of Dental Medicine Affiliate, Cambridge, MA, USA; ^6^Department of Pathology and Laboratory Medicine, University of Texas-Houston Medical School, Houston, TX, USA

Diabetes mellitus is a common chronic global disease affecting children and adolescents in developed as well as developing countries. Diabetes type I and type II are the major types of diabetes mellitus. The former is an autoimmune disorder, leading to pancreatic *β*-cell dysfunction and thus inadequate production of insulin. The latter arises from reduced sensitivity to insulin in the target tissues (i.e., skeletal muscle, liver, and adipose tissue) and later on insufficient insulin secretion. In both cases, the common result is sustained hyperglycemia. Uncontrolled hyperglycemia over time leads to deteriorating blood vessels supplying the body organs, leading to heart, eyes, kidneys, and nerves system damage. For instance, macrovascular (atherosclerotic) and microvascular (retinopathy and nephropathy) disorders are the leading causes of morbidity and mortality in diabetic patients [1]. We can appreciate then that diabetes is not a single disease but rather a combination of metabolic disorders [2].

According to the World Health Organization (WHO), more than 220 million people worldwide were diabetic in 2010 and this number will be doubled in 2040. The prevalence of diabetes is the highest in the Middle East, where the number of diabetic subjects reached 15.2 million in 2000 and it will almost be tripled within 30 years (from 15.2 million in 2000 to about and 42.6 million in 2030) [3].

Despite the great progress in synthetic drugs, herbal medicine has continued to be often utilized by people in most developed and developing nations. Furthermore, the popularity of herbal medicines preparations has increased worldwide in the past few decades, probably because of the sustainability of this medicine over the years. Moreover, herbal medicines are relatively inexpensive and are believed to be safer than synthetic drugs. Yet, care should be taken before the administration of any herbal medicine treatment [4].

This special issue provides a comprehensive overview on traditional herbal medicine including state-of-the-art description of traditional antidiabetic herbal medicine, their chemical composition, and activity. For this special issue, the editorial office received 22 papers and after rigorous peer review process 10 papers were accepted for publication. Out of 10 accepted papers, four articles are very informative high class review articles. All published article focused on the theme of this special issue which is utility of herbal medicines to act as complementary and alternative therapy for patients with diabetes and metabolic disorders. Four publications use* in vivo* rat model for their studies while in two publications the authors used* in vitro* model utilizing HepG2 cells. Toxicities of herbal supplements in humans have been reported, especially liver toxicity due to prolonged use of Kava, herbal sleep aid, and antidepressants.

There are four high class review articles published in this special issue. The review paper entitled “Natural Products Improving Hyperuricemia with Hepatorenal Dual Effects” by S. Hao et al. focuses on hyperuricemia (a risk factor for incident type II diabetes mellitus) treatment by natural products. Notably, classical medications used for treating high ureic acid such as allopurinol are highly toxic. The review article “*Euonymus alatus*: A Review on Its Phytochemistry and Antidiabetic Activity” by X. Zhai et al. summarized utility of an Asian plant* Euonymus alatus* in treating hyperglycemia, diabetic complications, and cancer. More than 100 chemicals including flavonoids, terpenoids, steroids, lignan, cardenolide, phenolic acid, and alkaloids are present in this plant extract. The review article “Antidiabetic Properties, Bioactive Constituents, and Other Therapeutic Effects of* Scoparia dulcis*” by G. Pamunuwa et al. discusses antidiabetic activities as well as antioxidant and anti-inflammatory properties of this herb. The review article “Applicability of Isolates and Fractions of Plant Extracts in Murine Models in Type II Diabetes: A Systematic Review” by G. Coelho et al. discusses both animal and human data available in effects of various herbs in lowering blood glucose level. All four excellent review articles are of valuable addition to current literature on utility of herbs in treating diabetes.

The original paper “The Hypoglycemic and Antioxidant Activity of Cress Seed and Cinnamon on Streptozotocin Induced Diabetes in Male Rats” by S. Qusti et al. demonstrates stimulation of pancreas in streptozotocin induced diabetic rats using 20% (w/w) garden cress seed* (Lepidium sativum)* and cinnamon methanol extracts. In another original paper “Evaluation of Hypoglycemic and Genotoxic Effect of Polyphenolic Bark Extract from* Quercus sideroxyla*” by M. Soto-Garcia et al., the authors evaluated potential hypoglycemic activity of bark of* Quercus sideroxyla* using rats where diabetes was induced by streptozotocin. Another* in vivo* study also using rat model “In Vivo Interrelationship between Insulin Resistance and Interferon Gamma Production: Protective and Therapeutic Effect of Berberine” by M. Mahmoud et al. is also very interesting as other publications have shown beneficial effect of berberine in insulin resistance.* Panax ginseng* antidiabetic activity was discussed in the original paper “Protective Effects of* Panax notoginseng* Saponins against High Glucose-Induced Oxidative Injury in Rat Retinal Capillary Endothelial Cells” by Y. Fan et al. The antioxidant property of another ginseng species is also discussed in this paper.

In two papers the authors used HepG2 cell as* in vitro* model for oxidative stress. “Effect of* Betula pendula* Leaf Extract on *α*-Glucosidase and Glutathione Level in Glucose-Induced Oxidative Stress” by K. Bljajic et al. demonstrates antioxidant effect of this herbal leaf. The authors used HepG2 cells to demonstrate* in vitro* the antioxidant effect of this leaf extract. “Diosgenin and 5-Methoxypsoralen Ameliorate Insulin Resistance through ER-*α*/PI3 K/Akt-Signaling Pathways in HepG2 Cells” by K. Fang et al. suggests a new strategy for diabetes type II treatment by targeting ER-mediated Akt signaling pathway.

## References

[1] Rivera A. L., Estañol B., Sentíes-Madrid H. (2016). Heart rate and systolic blood pressure variability in the time domain in patients with recent and long-standing diabetes mellitus. *PLoS ONE*.

[2] Zaid H., Antonescu C. N., Randhawa V. K., Klip A. (2008). Insulin action on glucose transporters through molecular switches, tracks and tethers. *Biochemical Journal*.

[3] Zaid H., Saad B., Mahdi A. A., Tamrakar A. K., Haddad P. S., Afifi F. U. (2015). Medicinal plants and natural active compounds for diabetes and/or obesity treatment. *Evidence-Based Complementary and Alternative Medicine*.

[4] Zaid H., Saad B., Watson R. R., Preedy V. R. (2013). State of the art of diabetes treatment in Greco-Arab and Islamic medicine. *Bioactive Food as Dietary Interventions for Diabetes*.

